# The Effects of Web-Based Patient Access to Laboratory Results in British Columbia: A Patient Survey on Comprehension and Anxiety

**DOI:** 10.2196/jmir.4350

**Published:** 2015-08-04

**Authors:** Geneviève Mák, Heather Smith Fowler, Chad Leaver, Simon Hagens, Jennifer Zelmer

**Affiliations:** ^1^ Social Research and Demonstration Corporation Ottawa, ON Canada; ^2^ Canada Health Infoway Toronto, ON Canada

**Keywords:** patient access to information, online access to laboratory results, consumer health solutions, personal health records, patient anxiety, patient comprehension, laboratory results

## Abstract

**Background:**

Web-based patient access to personal health information is limited but increasing in Canada and internationally.

**Objective:**

This exploratory study aimed to increase understanding of how Web-based access to laboratory test results in British Columbia (Canada), which has been broadly available since 2010, affects patients’ experiences.

**Methods:**

In November 2013, we surveyed adults in British Columbia who had had a laboratory test in the previous 12 months. Using a retrospective cohort design, we compared reported wait-time for results, test result comprehension, and anxiety levels of “service users” who had Web-based access to their test results (n=2047) with those of a general population panel that did not have Web-based access (n=1245).

**Results:**

The vast majority of service users (83.99%, 95% CI 82.31-85.67) said they received their results within “a few days”, compared to just over a third of the comparison group (37.84%, 95% CI 34.96-40.73). Most in both groups said they understood their test results, but the rate was lower for service users than the comparison group (75.55%, 95% CI 73.58-77.49 vs 84.69%, 95% CI 82.59-86.81). There was no significant difference between groups in levels of reported anxiety after receiving test results.

**Conclusions:**

While most patients who received their laboratory test results online reported little anxiety after receiving their results and were satisfied with the service, there may be opportunities to improve comprehension of results.

## Introduction

**Figure 1:**
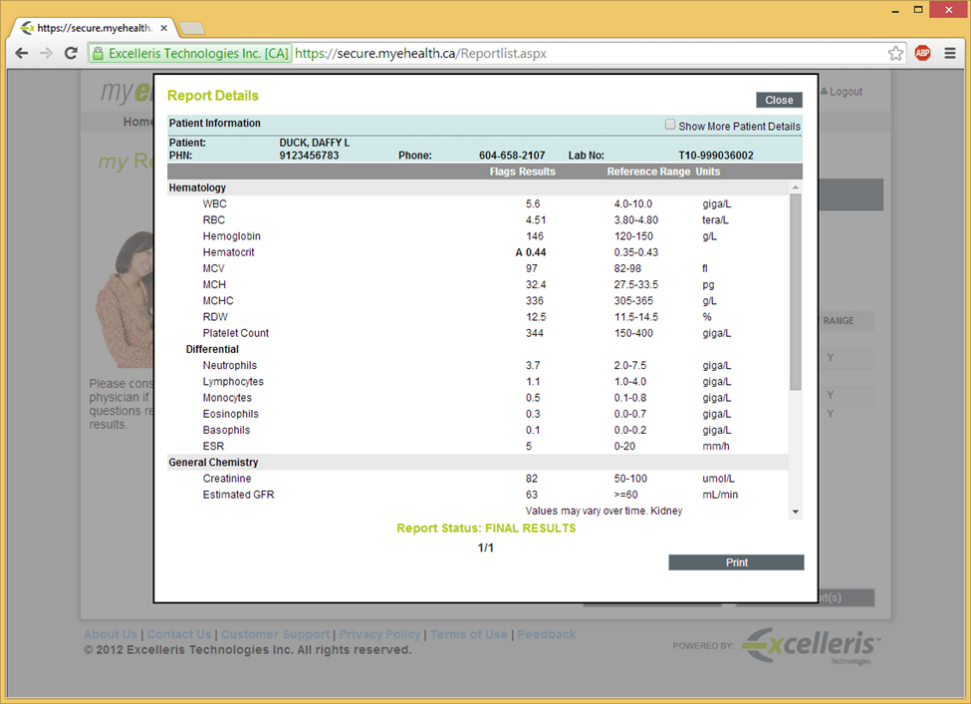
Screen capture of a sample lab report.

The use of consumer health solutions, including Web-based patient access to laboratory test results, is limited but expanding internationally [1]. In Canada, more than 8 in 10 adults express interest in use of such services, but in 2014, only about 6% said that they had online access to their laboratory test results. In British Columbia, availability was much higher than the national average (27% of those surveyed). Web-based access to laboratory test results has been available in most regions there since 2010 [2]. Subscribers to the direct lab access service in British Columbia create an account and register with secure passwords; access to the service is free. Patients often learn about the service and are provided secure access through the lab facility. After a lab test has been conducted, service users obtain Web-based access to their test results. The lab report is presented verbatim, as the ordering clinician would receive it, without additional information. Figure 1 presents a screenshot of a typical lab report, as would be available to patients. Ordering clinicians may contact their patients about the results or not, based on their clinical practice. Physicians do not have to subscribe to the service for patients to have access to lab test results.

As a relatively new technology, there is sparse literature about the benefits and risks of direct patient access to lab test results online, especially results that are abnormal or require follow-up with a health care provider [3]. For example, there is a lack of consensus on (1) best practices in direct patient notification of abnormal results, (2) whether patients will know what to do with the results, and (3) how they will react if they receive abnormal results online. Concerns about potential risks, such as patient anxiety or confusion, have been documented in the literature [4]. However, this association has not been found in the small body of research to date in this area. On the other hand, previous studies have identified advantages for patients who accessed their personal health records, such as improved quality of interactions with physicians, motivation to be better informed about and manage their own health [5-7], as well as a reduction of outpatient visits [8,9].

To our knowledge, this quasi-experimental study is the first to assess the effects of direct patient access to medical laboratory tests in Canada. Results presented here pertain to the experiences of British Columbia patients after seeing their lab test results online, focusing specifically on comprehension of test results and reported anxiety.

## Methods

### Data Sources

The service user and comparison cohorts were recruited separately. Service users were recruited from the subscriber database of the British Columbia service provider, specifically, from the approximately 15,000 subscribers who had given prior consent to be contacted for research purposes. Invitation emails were sent directly by the service provider in November 2013 to randomly selected subscribers (n=11,300) who were 18 years of age or older and had had a medical lab test conducted in the previous 12 months, the results of which they had accessed online. A pre-test of the survey was conducted among a small sample of participants in the online service users cohort (n=24). A total of 2047 service users fully completed the survey, for a response rate of 18.12%. While the bulk of the service users group consisted of participants who had first received their most recent test results online, some subscribers had first learned their most recent result in-person from their health care professional. The latter were omitted from some of the analyses to isolate the impact of receiving results online.

The comparison cohort was recruited in December 2013 from members of a general population panel maintained by Vision Critical, the composition of which is benchmarked against known census subgroups by age, gender, region, education, and income. The Vision Critical panel consists of approximately 130,000 panelists, of whom 15,000 reside in British Columbia. Each month, the panel fields a monthly survey to keep information about panelists current, to pre-screen panelists for specific study objectives, and to keep them actively engaged in the panel. In this case, the monthly screening tool served both to identify a potential comparison group and to target recruitment to match the online service users group as closely as possible on characteristics of age and gender. Two questions were used to pre-screen participants for our study: (1) “Have you had any medical laboratory tests conducted in the past 12 months?”, and (2) “If yes, how did you receive the results for your medical laboratory test(s): in-person, online, via mail, email, or over the phone?” The 20.60% of those screened who reported having received results online in the past 12 months were disqualified from our study. A total of 2762 panelists were recruited for the comparison group; these were randomly selected from demographic subgroups to balance the study cohorts. Of these, 1245 people fully completed the survey, resulting in a response rate of 45.08%.

Because of the relatively low response rate of service users, the results obtained are not considered representative of the broader population of online service users. The differences in response rates between the service users group and the comparison group may also have resulted in response bias (ie, nonresponse and voluntary response biases). Although statistical methods such as analytical weighting were used to balance observable characteristics of the two samples, differences in measured outcome indicators can be confounded with unobservable factors, such as familiarity with lab results or anxiety associated with inexperience. The results of this study should be therefore be interpreted with some caution. This study received ethics approval from Institutional Review Board Services (IRBS), Canada.

### Measures

Our survey included four sections: (1) self-reported health status and laboratory testing needs, (2) experience receiving the most recent lab test result, (3) perception of Web-based access to laboratory results (service users group only), and (4) sociodemographic characteristics and access to online consumer health solutions. Two questions were asked regarding respondents’ comprehension of lab test results: (1) “Was it clear if you needed to follow-up with your doctor? (Yes/No)”, and (2) “How confident are you that you fully understood your lab results?” on a scale of 1-10 (where 1=Not at all confident/10=Extremely confident).

The Global Anxiety-Visual Analog Scale (GA-VAS) (where “0” means “not at all anxious” and “100” means “extremely anxious”) was used because it has been reported to be useful and valid in assessing anxiety as a single construct (with many perceptible gradations) and as a measure of anxiety at a specific point in time, such as pre-operative anxiety [10].

### Analysis

Analyses were conducted using SPSS version 18.0. Preliminary analysis of our sample showed that the service users group had significantly more abnormal test results than the comparison group (633/1806, 35.1% vs 144/897, 16.1%). Since this variable was seen as key to patients’ overall experience, we weighted the sample on this variable in order to adjust for its effect (see Multimedia Appendix 1). Independent Student’s *t* tests for means or proportions of responses were used to assess differences between the two cohorts on sociodemographics, comprehension, and anxiety. For categorical responses, independent Student’s *t* tests were used for inference instead of distributional tests such as chi-square tests, in order to be able to detect any substantial differences across the specific response items. In addition, logistic regression techniques were used to examine the association between sociodemographic variables and comprehension.

It should be noted that missing responses were excluded from analyses. Also, in each table, *n* is calculated based on the actual number of respondents in the sample, while proportions are estimated with analytical weight.

## Results

### Sample Profile

Although the two cohorts were balanced in terms of age and gender, there were some statistically significant differences in other sociodemographic characteristics and health status (see Table 1). For example, the service users group included fewer participants who were born in Canada and who spoke English at home, more participants with university education, more urban participants, and more who made over CAN $100,000 in annual income. In terms of health status, participants from the service users group reported being in slightly poorer health than the comparison group and having undergone more medical lab tests (3 or more times) in the last 12 months.

Our overall sample included a high proportion of participants over age 55 who had a chronic illness and who were therefore more likely to require health care services. Women also outnumbered men, consistent with existing literature, which has found that more women subscribe to health portals than men [11,12].

Outcome analyses were conducted...at the time of the survey(1862/2047...respectively), using the weighting procedures described in Multimedia Appendix 1.

**Table 1:** Characteristics of survey participants.

		Service users group (n=2047)		Comparison group (n=1245)		*P*
		n	%	n	%	
**Gender**						
	Male	770	38.08	474	38.21	
	Female	1252	61.92	767	61.79	
**Age group, years**						
	18-34	193	9.75	115	9.41	
	35-54	566	28.60	347	28.27	
	55+	1220	61.65	765	62.32	
**Immigration status and language spoken at home**						
	Born in Canada: yes	1429	70.29	1043	83.84	<.001
	Language speak at home: English	1938	95.14	1231	98.91	<.001
**Income, CAN$**						
	<$50K	545	33.64	429	42.66	<.001
	$50K-$100k	626	38.64	369	36.74	
	≥$100K	449	27.72	207	20.60	<.001
**Education**						
	High school or less	164	8.18	155	12.76	<.001
	Some/Completed college	708	35.29	515	42.30	<.001
	Some university +	1134	56.53	547	44.93	<.001
**Region**						
	Greater Vancouver	1107	54.45	355	28.56	<.001
	Vancouver Island	442	21.74	287	23.12	
	BC Southern Interior	340	16.72	446	35.88	<.001
	BC Northern Interior	23	1.11	66	5.28	<.001
**Overall health (last 12 months)**						
	Excellent/Very good	868	42.48	552	44.42	
	Good	710	34.75	445	35.78	
	Fair/Poor	465	22.76	246	19.79	<.05
**Has a chronic health condition**						
	Yes	1213	60.35	727	58.82	
**Number of medical lab tests conducted (past 12 months)**						
	≥6 times	450	20.84	148	11.75	<.001
	3-5 times	834	40.55	412	33.05	<.001
	2 times	440	21.91	363	29.30	<.001
	1 time	323	16.70	322	25.90	<.001

### Wait Time to Receive Results

As expected, the wait time to receive lab test results was considerably shorter for the service users group, 87.50% (1624/1856) of whom first learned the result of their most recent lab test online. The majority of service users (83.99%, 95% CI 82.31-85.67) said they waited only “a few days” following their lab test before receiving their results, compared to just over a third of the comparison group (37.84%, 95% CI 34.96-40.73). Table 2 presents more information about the wait time to receive lab test results.

**Table 2:** Wait time to receive lab results.

	Service users group (n=1818), n (%) 95% CI	Comparison group (n=1087), n (%) 95% CI
Received results within a few days	1527 (83.99^a^) 82.31-85.67	411 (37.84) 34.96-40.73
Received results in about a week	209 (11.50) 10.03-12.96	335 (30.85 ^a^) 28.11-33.61
Received results in 1-2 weeks	56 (3.08) 2.29-3.89	221 (20.32 ^a^) 17.92-22.71
Received results between 2 and 4 weeks	16 (0.88) 0.45-1.31	85 (7.81 ^a^) 6.21-9.40
Received results in ˃4 weeks	10 (0.55) 0.21-0.89	35 (3.18 ^a^) 2.14-4.23

^a^ *P*<.001

### Comprehension of Lab Test Results

All those who knew their most recent test results (n=2990) were asked about their confidence in fully understanding the results, as measured by a score of 7 or higher on a scale of 1-10. The majority of both service users and the comparison group were confident they understood the test results themselves, but the percentage was lower for service users (see Table 3).

**Table 3:** Comprehension of lab results.

All who received results	Service users group (n=1852), n (%) 95% CI	Comparison group (n=1119), n (%) 95% CI
How confident are you that you fully understood your lab results (score=7-10)?	1399 (75.55) 73.58-77.49	948 (84.69^a^) 82.59-86.81

^a^ *P*<.001

To further explore what might influence patients’ comprehension of their lab results, we conducted a logistic regression using sociodemographic and health service–related variables, and adjusted the results for receipt of abnormal lab test results. The variable “first learned the result online” was used this time to assess how comprehension was influenced by the service itself and not simply by being a subscriber to the service more generally. As expected, first learning test results online was a significant *negative* predictor of comprehension, as were younger ages and lower levels of education (see Table 4).

**Table 4:** Very confident in fully understanding lab results (logistic regression; n=2796; 194 cases were excluded from analysis; % correct predicted values: 79.2%).

		95% CI for exp *b*			
		*B* (SE)	Lower	exp *b*	Upper
**Gender**					
	Female	-0.16 (0.10)	0.70	0.85	1.04
	Male (ref)	-	-	-	-
**Education**					
	High school or under	-0.72 (0.15)^a^	0.36	0.49	0.66
	Some/Completed college	0.24 (0.10)^b^	0.65	0.79	0.97
	Some university and + (ref)	-	-	-	-
**Age**					
	18-34	-0.48 (0.16)^a^	0.45	0.49	0.85
	35-54	-0.27 (0.11)^a^	0.62	0.77	0.95
	55 and + (ref)	-			
**First learned the result online**					
	Yes	-0.57 (0.10)^a^	0.47	0.57	0.69
	No (ref)	-	-	-	-
**Having a chronic condition**					
	Yes	-0.20 (0.10)^c^	0.68	0.82	1.00
	No (ref)	-	-	-	-
**Number of lab tests conducted (past 12 months)**					
	≥6 times	0.20 (0.13)	0.95	1.22	1.57
	≤5 (ref)	-	-	-	-
**Constant**		2.15 (0.13)^a^		8.58	

^a^ *P*<.001

^b^ *P*<.05

^c^ *P*<.01

### Anxiety

We also conducted between-group analyses on respondents’ reported level of anxiety *after* receiving their lab test results. Since the distribution of GA-VAS scores was positively skewed, we divided these into three categories: no reported anxiety (0), “low” anxiety (1-49), and “some” anxiety (50-100). To isolate the influence of Web-based access, we omitted from the analysis service users who had received the results of their most recent test in-person from their doctor or usual place of care.

We found no significant differences between service users and the comparison group in their level of anxiety following receipt of test results; as always, we adjusted for the effect of having a test result out of the normal range. As seen in Table 5, the majority of patients in both cohorts reported low or no anxiety after receiving test results. Table 5 also shows the results of analysis with a subgroup of participants in both cohorts who had a chronic health condition. Here, differences between the two cohorts emerged, such that service users reported being significantly less likely to be anxious at both ends of the scale (none and some).

**Table 5:** Anxiety after receiving lab test results.

	All		Subgroup with chronic condition	
	Subsample of service users who first learned results online (n=1478), n (%) 95% CI	Subsample comparison group who learned otherwise (n=1312), n (%) 95% CI	Subsample of service users who first learned results online (n=881), n (%) 95% CI	Subsample comparison group who learned otherwise (n=779), n (%) 95% CI
No anxiety (0)	540 (36.54) 34.08-38.99	447 (34.05) 31.49-36.62	310 (35.19^a^) 32.03-38.34	239 (30.64) 27.40-33.89
Low anxiety (1-49)	734 (49.66) 47.11-52.22	670 (51.07) 48.37-53.78	453 (51.42) 48.12-54.72	401 (51.47) 47.96-54.98
Some anxiety (50-100)	204 (13.80) 12.04-15.56	195 (14.87) 12.95-16.80	118 (13.39) 11.14-15.64	139 (17.89^a^) 15.19-20.58

^a^ *P*<.05

We also explored the link between anxiety and comprehension among service users specifically, to determine if individuals who first learned their results online *and* who reported lower levels of comprehension also reported more anxiety. To measure comprehension in relation to anxiety, respondents were asked how clear it was they needed to follow-up with their health care provider. As expected, service users who first learned the results of their most recent lab test online and who indicated it was clear they needed to follow-up were *less* likely to report no anxiety (38.30%, 95% CI 35.44-41.16) as those who reported not being clear about the need for follow-up (29.84%, 95% CI 24.69-34.98). The same pattern held at the other end of the anxiety spectrum. Overall, Table 6 shows that participants who were not clear on the need for follow-up were more likely to report being anxious.

**Table 6:** Anxiety level after receiving lab test results according to comprehension.

	Service users who first learned results online (n=1412)	
	Clear if you need to follow-up? Yes (n=1107) n (%) 95% CI	Clear if you need to follow-up? No (n=305) n (%) 95% CI
No anxiety (0)	424 (38.30^a^) 35.44-41.16	91 (29.84) 24.69-34.98
Low (1-49)	551 (49.77) 46.83-52.72	153 (50.16) 44.54-55.78
Some (50-100)	132 (11.92) 10.01-13.83	61 (20.00^a^) 15.50-24.50

^a^ *P*<.001

## Discussion

### Principal Findings

Our findings suggest that patient experience overall can be improved by the availability of Web-based lab results, but with important caveats. Service users were more likely to report a shorter wait for test results and high levels of satisfaction with the online service. There was no overall difference in post-result anxiety levels between those who saw results online and those who received results in other ways (eg, by mail or telephone), although among the subset of patients with chronic conditions, service users were less likely to report anxiety. However, service users were *more* likely to report lower comprehension of lab test results than the comparison group, and there was a significant correlation between anxiety and lower comprehension. This is not surprising, given that the format of lab results provided by the Web-based platform is the same as that provided to ordering clinicians, with no additional contextual or explanatory information for patients.

Our study results support Pyper et al’s call for more information and tools to help patients understand and interpret their health information [5]. A range of tools has been suggested in the literature, including a glossary, integration with other health records, and patient education/information support.

### Limitations

Although our comparison sample was recruited from a general population panel, differences between the service users group and the comparison group—including the rate of abnormal test results—somewhat limits the study’s external validity. The service user group also had a lower response rate at 18%, which may indicate a possible response bias. Both cohorts were also recruited online, so our findings may not apply to the rapidly diminishing proportion of the population that does not have Internet access and a degree of digital literacy.

We also do not know much about the sequence and timing involved in obtaining test results by different means, and follow-up information and support. For example, we could not differentiate between tests conducted for diagnostic purposes versus for monitoring a previously known health condition. Additional clinical information would have provided a more focused interpretation of results about the patient experience. While we deliberately focused many of our survey questions on the most recent test to enhance precision of responses (ie, content validity) and their reliability, patients’ most recent tests may not reflect their typical experience with lab tests or with direct lab access in general. Moreover, we did not focus our study on the experiences of patients with abnormal test results, possibly diluting any negative effects of Web-based access on anxiety and comprehension, if they exist.

Finally, while we balanced our two cohorts as closely as possible and weighted the comparison cohort to take into account the type of test result received (ie, in the normal range or not), the quasi-experimental design of our study limits our ability to attribute observed differences to the intervention with the same degree of confidence as with random assignment to treatment, had that been possible.

### Conclusions

Laboratory tests are among the most common interventions in modern health systems, and effective communication of test results and required follow-up is a priority for research. As jurisdictions around the world move toward widespread adoption of digital health technologies for patients, better understanding is needed of the effects of such services on both patients and health care practice. This study explored these issues in relation to one such technology—direct patient access to Web-based lab test results—currently in use in British Columbia, Canada. While exploratory, our study supports the emerging literature suggesting that personal health records are positively received by patients [13,14]. It also supports a recent systematic review that found access to health records reduced or had no effect on anxiety [15]. In our case, we found no differences between service users and a comparison group on reported anxiety after receiving test results, although we did find that this differed by level of comprehension. While this study contributes to understanding the extent and nature of benefits and risks associated with direct lab access, important questions remain for future research about the mechanisms by which these benefits are achieved, how such benefits can be optimized in different health care contexts and for different subgroups of the population, and the specific experiences of patients who receive abnormal test results.

## Appendix

Multimedia Appendix 1 Note on the weight construction.

## Abbreviations

GA-VAS: Global Anxiety-Visual Analog Scale

## References

[1] Zelmer J, Hagens S (2014). Understanding the gap between desire for and use of consumer health solutions. Healthc Pap.

[2] Harris Decima (2014). Annual tracking survey of the general population. Canada Health Infoway.

[3] Malone B (2012). Direct Patient Access to Lab Results: Are Labs Ready to Respond to Patient Requests?.

[4] Giardina D, Singh H (2011). Should patients get direct access to their laboratory test results?: An answer with many questions. JAMA.

[5] Pyper C, Amery J, Watson M, Crook C (2004). Patients' experiences when accessing their on-line electronic patient records in primary care. Br J Gen Pract.

[6] Archer N, Fevrier-Thomas U, Lokker C, McKibbon KA, Straus SE (2011). Personal health records: a scoping review. J Am Med Inform Assoc.

[7] Siteman E, Businger A, Gandhi T, Grant R, Poon E, Schnipper J, Volk LA, Wald JS, Middleton B (2006). Clinicians recognize value of patient review of their electronic health record data. AMIA Annu Symp Proc.

[8] Ammenwerth E, Schnell-Inderst P, Hoerbst A (2012). The impact of electronic patient portals on patient care: a systematic review of controlled trials. J Med Internet Res.

[9] Chen C, Garrido T, Chock D, Okawa G, Liang L (2009). The Kaiser Permanente Electronic Health Record: transforming and streamlining modalities of care. Health Aff (Millwood).

[10] Kindler CH, Harms C, Amsler F, Ihde-Scholl T, Scheidegger D (2000). The visual analog scale allows effective measurement of preoperative anxiety and detection of patients' anesthetic concerns. Anesth Analg.

[11] Hassol A, Walker JM, Kidder D, Rokita K, Young D, Pierdon S, Deitz D, Kuck S, Ortiz E (2004). Patient experiences and attitudes about access to a patient electronic health care record and linked web messaging. J Am Med Inform Assoc.

[12] Leveille SG, Walker J, Ralston JD, Ross SE, Elmore JG, Delbanco T (2012). Evaluating the impact of patients' online access to doctors' visit notes: designing and executing the OpenNotes project. BMC Med Inform Decis Mak.

[13] Lin C, Wittevrongel L, Moore L, Beaty BL, Ross SE (2005). An Internet-based patient-provider communication system: randomized controlled trial. J Med Internet Res.

[14] Ross SE, Moore LA, Earnest MA, Wittevrongel L, Lin C (2004). Providing a web-based online medical record with electronic communication capabilities to patients with congestive heart failure: randomized trial. J Med Internet Res.

[15] Giardina T, Menon S, Parrish D (2014). Patient access to medical records and healthcare outcomes: a systematic review. JAMIA.

