# Supplementary material for: The Effects of Web-Based Patient Access to Laboratory Results in British Columbia: A Patient Survey on Comprehension and Anxiety
Source: J Med Internet Res. 2015 Aug 4;17(8):e191. doi: 10.2196/jmir.4350 (PMC4705365; doi:10.2196/jmir.4350)
Supplement: Multimedia Appendix 1 [file jmir_v17i8e191_app1.pdf]

Appendix A: Multimedia Appendix 1. Note on the weight construction.

Question 9 asked whether the respondent knew their most recent lab test, and if the response was "yes", the respondent would be followed up with Question 17 asking whether the recent test result was considered within the normal range.

The distribution of answers are presented in Table B1. Without any adjustment or analytical weight, 57.3% and 30.9% of the service users group sample reported that their recent lab test result was within and out of the normal range, respectively. Among the comparison group sample, 60.5% and 11.6% of respondent reported that their recent lab test result was within and out of the normal range, respectively (Table B2).

In order to balance these differences in the responses to Questions 9 and 17, analytical weighting was created (Table B3) and applied to the outcome analysis. The weighted distribution of responses to Questions 9 and 17 among the comparison group match exactly that of the service users group. In other words, after applying the analytical weight, the two samples were completely balanced regarding the composition of receiving lab test within and out of the normal range.

Table 1. Table B1 Frequencies of individuals (per cohort) who know the results of their most recent test and had an abnormal result

	Service Users Group	Comparison Group
**Q9: Do you know the result of your most recent lab test?**		
Missing	3	4
No	182	326
Yes	1862	915
**Q17: Was your most recent test considered within the normal range?**		
Missing	56	18
Yes	1173	753
No	633	144

Table 2. Table B2 Proportion in each group in each cohort who received an abnormal result

Service Users Group

Comparison Group

Q9 = missing or Q17 missing

t1 = 3+56/2047 = 0.0288

a1 = 4+18/1245 = 0.0177

Q9 = No

t2 = 182/2047 = 0.0889

a2 = 326/1245 = 0.2618

Q9 = Yes & Q17 = Yes

t3 = 1173/2047 = 0.573

a3 = 753/1245 = 0.6048

Q9 = Yes & Q17 = No

t4 = 633/2047 = 0.3092

a4 = 144/1245 = 0.1157

Table 3. Table B3 Weight calculation in each cohort

	Service Users	Comparison
Q9 = missing or Q17 missing	w = 1	w = 0.0288/0.0177 = 1.631
Q9 = No	w = 1	w = 0.0889/0.2618 = 0.3396
Q9 = Yes & Q17 = Yes	w = 1	w = 0.573/0.6048 = 0.9475
Q9 = Yes & Q17 = No	w = 1	w = 0.3092/0.1157 = 2.6736
